# Methylseleninic Acid Sensitizes Notch3-Activated OVCA429 Ovarian Cancer Cells to Carboplatin

**DOI:** 10.1371/journal.pone.0101664

**Published:** 2014-07-10

**Authors:** Tiffany J. Tzeng, Lei Cao, YangXin Fu, Huawei Zeng, Wen-Hsing Cheng

**Affiliations:** 1 Department of Nutrition and Food Science, University of Maryland, College Park, Maryland, United States of America; 2 Department of Food Science, Nutrition and Health Promotion, Mississippi State University, Mississippi State, Mississippi, United States of America; 3 Department of Oncology, University of Alberta, Edmonton, Alberta, Canada; 4 USDA, Agriculture Research Service, Grand Forks Human Research Center, Grand Forks, North Dakota, United States of America; Institut Jacques Monod, France

## Abstract

Ovarian cancer, the deadliest of gynecologic cancers, is usually not diagnosed until advanced stages. Although carboplatin has been popular for treating ovarian cancer for decades, patients eventually develop resistance to this platinum-containing drug. Expression of neurogenic locus notch homolog 3 (Notch3) is associated with chemoresistance and poor overall survival in ovarian cancer patients. Overexpression of NICD3 (the constitutively active form of Notch3) in OVCA429 ovarian cancer cells (OVCA429/NICD3) renders them resistance to carboplatin treatment compared to OVCA429/pCEG cells expressing an empty vector. We have previously shown that methylseleninic acid (MSeA) induces oxidative stress and activates ataxia-telangiectasia mutated and DNA-dependent protein kinase in cancer cells. Here we tested the hypothesis that MSeA and carboplatin exerted a synthetic lethal effect on OVCA429/NICD3 cells. Co-treatment with MSeA synergistically sensitized OVCA429/NICD3 but not OVCA429/pCEG cells to the killing by carboplatin. This synergism was associated with a cell cycle exit at the G2/M phase and the induction of NICD3 target gene *HES1*. Treatment of *N*-acetyl cysteine or inhibitors of the above two kinases did not directly impact on the synergism in OVCA429/NICD3 cells. Taken together, these results suggest that the efficacy of carboplatin in the treatment of high grade ovarian carcinoma can be enhanced by a combinational therapy with MSeA.

## Introduction

Results from geographic, animal and clinical studies strongly point to a positive association between selenium and chemoprevention [1]–[3]. Nonetheless, supranutritional intake of dietary selenium in the form of selenomethionine does not prevent prostate cancer [4]. Among the many selenium compounds, methylseleninic acid (MSeA) has been demonstrated to be exceptionally effective in counteracting prostate, pancreatic and breast cancers in mice [5]–[8]. The efficacy of selenium chemoprevention also depends on baseline selenium status and genetic background [9]. MSeA is metabolized to methylselenol, eventually resulting in the formation of selenium dioxide, superoxide anion, and hydrogen peroxide [10], [11]. Furthermore, MSeA can activate ataxia-telangiectasia mutated (ATM) and the catalytic subunit of DNA-dependent protein kinase (DNA-PK_cs_), two critical DNA damage response kinases, in cancer cells [12]–[14].

Mammals express four neurogenic locus notch homolog (Notch) family proteins during tumorigenesis and embryogenesis [15], [16]. Unlike many other signaling molecules, activation of the Notch pathway does not require secondary messengers for amplification [17]. Upon ligand binding to the N-terminal EGF-repeat region, the Notch transmembrane receptor undergoes a series of proteolytic cleavages by tumor necrosis factor-α-converting enzyme, metalloprotease, and γ-secretase [18]. γ-Secretase cleavage releases the Notch intraceullular domain (NICD) into cytoplasm, followed by translocation to the nucleus for transactivation of a spectrum of genes involved in tumor development and progression [19]–[21]. Thus, targeting Notch is considered promising for the improvement of platinum-based chemotherapy [22].

Platinum compounds have been approved by US Food and Drug Administration for cancer treatment since 1979. With reduced side effects, carboplatin [*cis*-diammine (1,1-cyclobutanedicarboxylato) platinum(II)] is the most effective second generation platinum compound for treatment of ovarian and testicular cancer [23], [24]. Carboplatin alkylates DNA bases and forms monoadducts, the majority of which eventually are converted into DNA crosslinks [25], [26]. Interestingly, cells uptake carboplatin in a manner depending on a copper transporter, CTR1 [27]–[29].

Most ovarian cancer patients are diagnosed at an advanced stage because of the lack of validated screening tests. Despite of being the most effective drug to treat ovarian cancer, resistance to carboplatin develops in some high grade tumors. Aberrant Notch activation is strongly associated with carboplatin resistance [22], [30], [31]. In particular, Notch3 is overexpressed in 66% of high grade ovarian carcinoma [32], 22% of which at stages II–IV exhibit altered Notch signaling [33]. Given such findings, the studies reported herein were designed to determine whether MSeA could sensitize Notch3-activated ovarian cancer cells to carboplatin treatment.

## Materials and Methods

### Cell culture and chemicals

OVCA429 cells were isolated from a patient with late stage, cisplatin-resistant ovarian carcinoma. OVCA429/pCEG cells carrying a green fluorescence protein empty vector and OVCA429/NICD3 cells constitutively expressing green fluorescence protein tagged with NICD3 were sorted by a fluorescence-activated cell sorter and maintained in RPMI 1640 medium (Mediatech Inc, Herndon, VA) supplemented with 10% heat-inactivated fetal bovine serum and 100 U/mL penicillin and streptomycin at 37°C in a 5% CO_2_ incubator [34]. *N*-acetyl cysteine (NAC) and MSeA (Sigma-Aldrich, St. Louis, MO) were dissolved in phosphate-buffered saline. NAC is an antioxidant that mainly abolishes hydrogen peroxide. Carboplatin (Enzo Life Sciences, Farmingdale, NY) was dissolved in water. KU 60019 and NU 7026 (Tocris, Ellisville, MO) were dissolved in dimethyl sulfoxide, and were ATP-competitive, selective chemical inhibitors of the kinase activity of ATM and DNA-PK_cs_ [35], respectively.

### Cell viability

Sulforhodamine B (SRB) colorimetric assays were performed as described previously [36]. Briefly, cells were seeded (10^5^ cells/well) in 96-well plates and allowed for attachment overnight prior to drug treatment. Then, cells were fixed with 10% trichloroacetic acid for 1 h at 4 °C, washed 5 times with water and air dried, and then stained with 0.4% SRB in 1% acetic acid for 20 minutes at room temperature. Unbound dye was removed by gently washing the cells 5 times with 1% acetic acid. After being air dried, cells were incubated with 200 µL of tris(hydroxymethyl)aminomethane buffer (pH 10.5, 10 mmol/L) for 30 minutes at 37 °C. The optical density was measured by a plate reader (BMG LabTech, Cary, NC) at 492 nm. Percent cell viability was calculated using the formula [36]:

% cell viability =  [(meanOD_sample_ − meanOD_day 0_)/(meanOD_neg control_ − meanOD_day 0_)] ×100%"

Cell viability was further analyzed by calculating combination index (CI) values with the Calcusyn software (Biosoft) based on the theorem of Chou-Talalay. The synergy CI scale is from 1 to 0 and the antagonism is from 1 to infinity [37].

### Immunofluorescence

Immunofluorescent analyses of ATM phosphorylation on Ser-1981 (pATMS1981), DNA-PK_cs_ phosphorylation on Ser-2056 (pDNA-PK_cs_S2056) and H2AX phosphorylation on Ser-139 (γH2AX) were performed as described previously [14], [38], [39]. All images were taken under the same parameters of brightness, contrast, and exposure time and processed by deconvolution using AxioVision Release 4.7.2.0 coupled to a Zeiss Axio Observer Z1m fluorescence microscope (Zeiss, Thornwood, NY). Five pictures were randomly taken from each slide (n  =  3).

### Cell cycle and selenium analyses

Flow cytometric analyses of cell cycle were performed as described previously [12]. Cells were analyzed by a FACScalibur cytometer with CELLQuest program (Becton Dickinson, San Jose, CA). ModFit LT (Version 3.0, Verity Software House, Topsham, ME) was applied for cell cycle analysis on overlaid histograms. Intracellular selenium concentrations were determined as described previously [40], [41].

### RNA isolation and quantitative RT-PCR (qRT-PCR)

Total RNA was isolated by using chloroform for phase separation, isopropanol for RNA precipitation, 75% ethanol for RNA wash, RNase-free water for RNA resuspension, and DNase-treated before cDNA was synthesized using High-Capacity cDNA Reverse Transcription kit (Life Technologya) in the presence of RNase inhibitor. qRT-PCR was carried out using the SYBR Green method on an Applied Biosystems 7500 Fast Real-time (Applied Biosystems, Foster Ciet, CA). Sequences of the primers for qPCR are listed in Table S1 in File S1.

### Statistics

These data were analyzed by using SAS 9.0 (SAS Institute Inc., Cary, NC). Two-tailed student's *t*-test was applied to determine statistical significance (*p* < 0.05) between the treatment and the respective control groups.

## Results

### Synergistic lethality of MSeA and carboplatin in OVCA429/NICD3 cells

**Figure 1:**
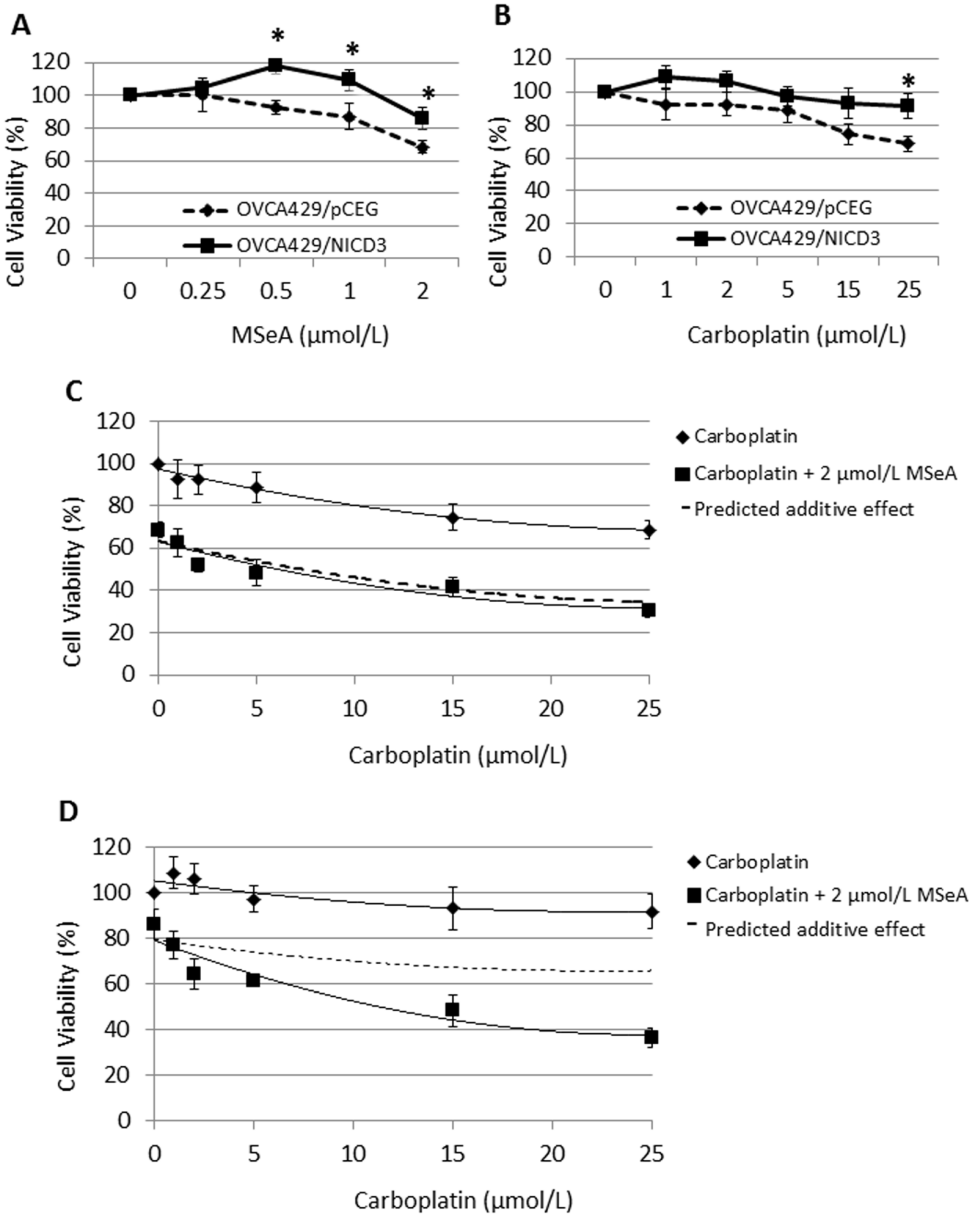
Synergistic effect of MSeA and carboplatin on the killing of OVCA429/NICD3 cells. OVCA429/pCEG and OVCA429/NICD3 cancer cells were treated with a gradient concentration of MSeA (*A*) or carboplatin (*B*) for 2 days. *, *p* < 0.05, compare to OVCA429/pCEG cells. OVCA429/pCEG cells (*C*) and OVCA429/NICD3 cells (*D*) were treated with carboplatin (0–25 µmol/L) in the absence or presence of MSeA (2 µmol/L) for 2 days. Values are mean ± S.E.M. (n  =  3). Dashed lines predict the additive effect of MSeA and carboplatin.

Ovarian carcinomas expressing NICD3 are resistant to platinum therapeutic agents [22], [30], [31]. We have previously shown that MSeA treatment (LD_50_, 4 µmol/L) kills HCT116 colorectal, PC-3 prostate and U-2 OS osteosarcoma cells in association with reactive oxygen species (ROS), ATM and DNA-PK_cs_ [12], [13]. Because ROS are also implicated in Notch3 signaling pathway [42], [43], we tested the hypothesis that MSeA could repress the desensitization of OVCA429/NICD3 ovarian cancer cells to carboplatin. Results from SRB survival assays demonstrated that MSeA (0.25–2 µmol/L, Figure 1A) or carboplatin (1–25 µmol/L, Figure 1B) alone dose-dependently killed more OVCA429/pCEG than OVCA429/NICD3 cells. Results from combinational treatment (Table 1) suggested that MSeA (2 µmol/L) and carboplatin (1-25 µmol/L) synergistically sensitized OVCA429/NICD3 cells (Figure 1D) but not OVCA429/pCEG cells (Figure 1C). Further CI analyses confirmed strong synergism between MSeA (2 µmol/L) and carboplatin (1–25 µmol/L) in OVCA429/NICD3 cells (Table 2). The synergism was linearly enhanced as carboplatin concentrations increased. Interestingly, based on CI values (Table 2), moderate to strong antagonism occurred after co-treatment with MSeA at 2 µmol/L in OVCA429/pCEG cells and ≤ 1 µmol/L in some of the OVCA429/NICD3 cells. In particular, the MSeA (2 µmol/L) and carboplatin (25 µmol/L) co-treatment sensitized the refractory OVCA429/NICD3 cells to an extent reminiscent of that in OVCA429/pCEG cells (36.2 vs. 30.2% survival). Taken together, MSeA can synergistically sensitize Notch3-activated OVCA ovarian cancer cells to the traditional carboplatin treatment at pharmacologically achievable concentrations.

**Table 1:** Sensitivity of OVCA429/pCEG and OVCA429/NICD3 ovarian cancer cells to MSeA and carboplatin treatment.

OVCA429/pCEG						
MSeA (µmol/L)	Carboplatin (µmol/L)					
	0	1	2	5	15	25
0	100	92.5±9.4	92.2±7.1	88.8±7.2	74.4±6.1^*^	68.4±4.3^*^
0.25	100.2±10.2	90.0±5.4	89.9±7.9	93.5±13.8	75.3±7.2	66.1±11.3
0.5	92.7±4.4	94.1±6.4	89.9±7.5	84.2±8.6	75.2±7.5	63.5±9.6
1	87.1±8.2	82.3±6.5	77.9±6.6	76.5±4.0	61.4±3.6	47.6±0.7^#*^
2	68.5±3.5^#^	62.6±6.4	51.7±3.1^#*^	48.3±6.1^#*^	41.3±4.6^#*^	30.3±3.2^#*^

OVCA429/NICD3						
MSeA (µmol/L)	Carboplatin (µmol/L)					
	0	1	2	5	15	25
0	100	108.5±7.0	106.0±6.4	97.1±5.7	93.2±9.3	91.7±7.5
0.25	104.6±3.5	101.7±5.2	101.8±5.9	105.2±6.8	98.3±5.2	96.5±10.6
0.5	117.8±4.8^#^	110.3±5.2	118.3±4.6	104.7±4.9	95.8±6.0^*^	83.6±9.8^*^
1	109.3±6.5	103.7±4.0	113.3±3.6	98.8±6.6	88.5±5.5	75.4±8.8^*^
2	86.1±6.8	76.9±6.1^#^	64.2±6.6^#^	61.2±1.2^#*^	48.2±6.9^#*^	36.2±4.4^#*^

Cells were cultured in 96-well plates and treated with MSeA and carboplatin at the indicated concentration for 2 days. Cell viability was determined by SRB assay. The condition without MSeA or carboplatin treatment was set as 100%. Values are mean ± S.E.M. (n  =  3). ^#^, *p* < 0.05, compared to no MSeA treatment. *, *p* < 0.05, compared to no carboplatin treatment.

**Table 2:** Combination index (CI) values for MSeA and carboplatin treatment in OVCA429/pCEG and OVCA429/NICD3 ovarian cancer cells.

OVCA429/pCEG					
MSeA (µmol/L)	Carboplatin (µmol/L)				
	1	2	5	15	25
0.25	0.61	0.98	>3.3	1.12	0.90
0.5	1.46	1.20	1.21	1.31	0.96
1	0.93	0.94	1.13	1.04	0.91
2	1.51	1.47	1.47	1.48	1.41

OVCA429/NICD3					
MSeA (µmol/L)	Carboplatin (µmol/L)				
	1	2	5	15	25
0.25	0.36	0.63	2.64	2.13	2.66
0.5	2.80	>3.3	2.55	1.53	0.59
1	0.96	>3.3	1.13	0.75	0.34
2	0.26	0.16	0.15	0.09	0.05

Based on a refined description made by an inventor of the theorem of Chou-Talalay, the following descriptions of CI values are employed: <0.3, strong synergism; 0.3–0.7, synergism; 0.7–0.9, moderate or slight synergism; 0.9–1.1, nearly additive; 1.1–1.45, slight or moderate antagonism; 1.45–3.3, antagonism; >3.3, strong antagonism [37]. Cell viability and treatment are as described in Table 1.

### Cell cycle analysis of OVCA429/pCEG and OVCA429/NICD3 cells co-treated with MSeA and carboplatin

In the absence of MSeA and carboplatin, there were greater G2/M and less G1 and S populations (*p* < 0.05) in OVCA429/NICD3 than in OVCA429/pCEG cells (Table 3). Two days after co-treatment of MSeA (2 µmol/L) and carboplatin (5 µmol/L), S and G2/M population was significantly decreased (*p* <0.05) in OVCA429/pCEG and OVCA429/NICD3 cells, respectively. OVCA429/pCEG and OVCA429/NICD3 cells comparably displayed a time-dependent induction of DNA fragmentation after the co-treatment as evidenced by sub-G1 populations. These results suggest that the co-treatment differentially target the S phase in OVCA429/pCEG cells and the G2/M phase in OVCA429/NICD3 cells.

**Table 3:** Flow cytometric analyses of the percent G1, S, and G2/M OVCA429/pCEG and OVCA429/NICD3 cells co-treated with MSeA (2 µmol/L) and carboplatin (5 µmol/L) for 1 or 2 days.

	Day		
	0	1	2
*Sub G1, %*			
OVCA429/pCEG	0.7±0.1*	2.2±0.4^#*^	8.9±1.0^#^
OVCA429/NICD3	1.4±0.1	3.4±0.3^#^	9.1±0.9^#^
*G1, %*			
OVCA429/pCEG	32.7±1.0*	34.7±2.7*	34.0±2.7*
OVCA429/NICD3	13.4±0.6	12.7±0.8	15.7±0.3^#^
*S, %*			
OVCA429/pCEG	27.1±3.1*	25.1±2.7*	18.2±1.2^#*^
OVCA429/NICD3	12.3±0.6	13.9±0.7	12.4±0.8
*G2/M, %*			
OVCA429/pCEG	39.6±2.5*	38.0±0.5*	38.9±2.5*
OVCA429/NICD3	72.9±0.7	70.0±0.8	62.7±1.1^#^

Values are mean ± S.E.M. (n  =  3). *, *p* < 0.05, compared to OVCA429/NICD3 cells. ^#^, *p* < 0.05, compared to Day 0.

### Effect of NAC, KU 60019, and NU 7026 on the sensitivity of OVCA429/pCEG and OVCA429/NICD3 cells to the MSeA and carboplatin co-treatment

**Figure 2:**
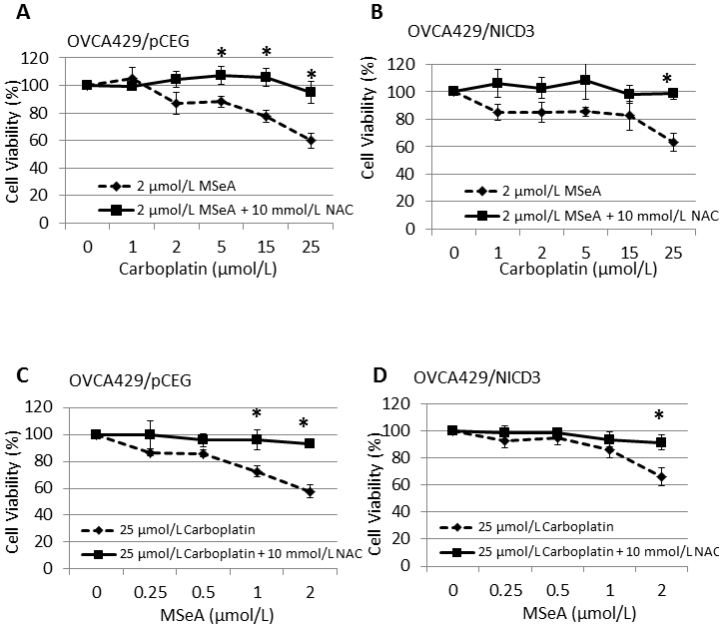
The effect of NAC on the sensitivity of OVCA429/pCEG and OVCA429/NICD3 cells to MSeA and carboplatin co-treatment. OVCA429/pCEG (*A*, *C*) and OVCA429/NICD3 (*B*, *D*) cells were treated with MSeA and a gradient concentration of carboplatin (*A*, *B*) or carboplatin and a gradient concentration of MSeA (*C*, *D*) in the presence or absence of NAC. Cell viability was determined by SRB assay. Viability of the cells without carboplatin (*A*, *B*) or MSeA (*C*, *D*) treatment was set as 100%. Values are mean ± S.E.M. (n  =  3). *, *p* < 0.05, compared to MSeA or carboplatin only treatment.

**Figure 3:**
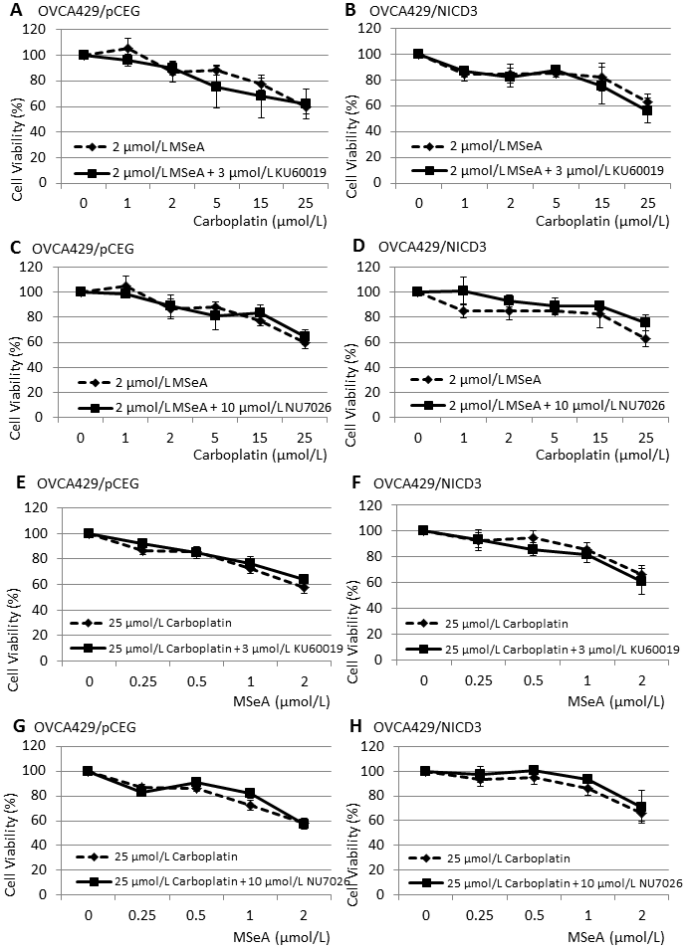
The effect of KU 60019 and NU 7026 on the sensitivity of OVCA429/pCEG and OVCA429/NICD3 cells to MSeA and carboplatin co-treatment. Cells were treated with MSeA and a gradient of carboplatin (*A*–*D*) or carboplatin and a gradient concentration of MSeA (*E*–*H*) in the presence or absence of KU 60019 (*A*, *B*, *E*, *F*) and NU 7026 (*C*, *D*, *G*, *H*). Cell viability was determined by SRB assay. Values are mean ± S.E.M. (n  =  3).

Next, we determined whether redox status and the kinase activities of ATM and DNA-PK_cs_ were involved in the sensitivity of OVCA429/pCEG and OVCA429/NICD3 cells to the MSeA and carboplatin co-treatment. In the presence of NAC (10 mmol/L), the killing effect of MSeA and carboplatin was greatly alleviated in both cell lines (Figures 2A–2D). In contrast, the presence of KU 60019 (3 µmol/L) or NU 7026 (10 µmol/L) did not alter the sensitivity of OVCA429/pCEG or OVCA429/NICD3 cells to gradient concentrations of MSeA and carboplatin co-treatment (Figure 3). These results suggest that the induction of ROS, but not ATM or DNA-PK_cs_ kinase activities, is involved in the killing effect of MSeA and carboplatin co-treatment.

### Effect of MSeA and carboplatin on the mRNA expression of Notch target genes in OVCA429/pCEG and OVCA429/NICD3 cells

**Figure 4:**
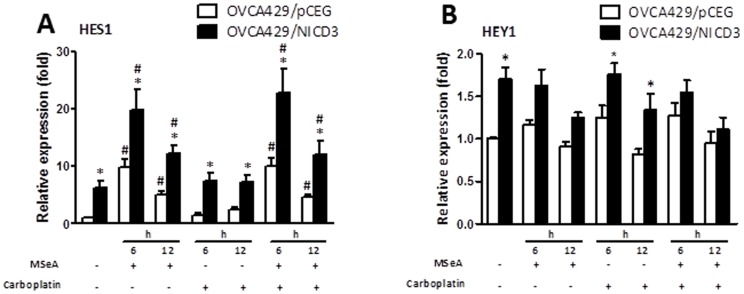
The effect of MSeA and carboplatin on the mRNA expression of *HES1* and *HEY1* in OVCA429/pCEG and OVCA429/NICD3 cells. The mRNA levels were normalized by those of β-actin and presented as fold changes relative to the OVCA429/pCEG cells without MSeA (2 µmol/L) and carboplatin (25 µmol/L) treatment. Values are mean ± S.E.M. (n  =  3). *, *p* < 0.05, compared to OVCA429/pCEG cells. #, *p* < 0.05, compared to cells without MSeA and carboplatin treatment.

We next determined whether the mRNA expression of Notch target genes can be altered by MSeA and carboplatin treatment. As expected, *HES1 and HEY1*, classical Notch target genes, were up-regulated in OVCA429/NICD3 cells (Figure 4). *HES1* mRNA expression was increased (*p* < 0.05) 6 and 12 h after MSeA treatment in both OVCA429/pCEG and OVCA429/NICD3 cells, the fold-induction of which was greater in the former than the latter. The MSeA-induced *HES1* mRNA expression subsided at 12 h. In contrast, carboplatin treatment resulted in modest and late induction of *HES1* expression. However, *HEY1* mRNA expression was not affected by MSeA or carboplatin treatment in both OVCA429/pCEG and OVCA429/NICD3 cells. Altogether, MSeA induces the mRNA expression of *HES1* independent of carboplatin or NICD3 expression.

### Effect of MSeA and carboplatin co-treatment on the formation of pATMS1981, pDNA-PK_cs_S2056 and γH2AX in OVCA429/pCEG and OVCA429/NICD3 cells

We next assessed cellular DNA damage response to the co-treatment. At 24 h, pDNA-PK_cs_S2056 level rose significantly (*p* < 0.05) and the induction could be completely reversed in the presence of NU 7026 in both OVCA429/pCEG and OVCA429/NICD3 cells (Figure S1A in File S1). The presence of NAC attenuated pDNA-PK_cs_S2056 expression in OVCA429/pCEG but not in OVCA429/NICD3 cells. On the other hand, pATMS1981 expression was induced (*p* < 0.05) by the co-treatment in OVCA429/pCEG but not in OVCA429/NICD3 cells (Figure S1B in File S1). The induction of pATMS1981 expression was suppressed in the presence of KU 60019 or NAC. The MSeA and carboplatin co-treatment did not induce γH2AX formation (Figure S2 in File S1), which is consistent with previous reports showing that carboplatin treatment, even at a very high dose (100 µmol/L), only slightly induces γH2AX formation in OVCAR-3 ovarian cancer cells [44], [45]. Altogether, pDNA-PK_cs_S2056 and pATMS1981 respond differentially to MSeA and carboplatin co-treatment and do not directly correlated with the killing effect in OVCA429/pCEG and OVCA429/NICD3 cells.

## Discussion

Results from the present study demonstrate that MSeA can synergistically enhance the efficacy of carboplatin in the killing of OVCA429/NICD3 ovarian cancer cells, suggesting a new strategy to treatment of high grade ovarian carcinoma exhibiting Notch3 activation. Such synergistic effect is likely attributed to the modulation of NICD3 transactivation events regulated by MSeA and carboplatin. Carboplatin treatment or the expression of NICD3 does not affect selenium contents in MSeA-treated cells (OVCA429/pCEG cells, 21.5±2.6 vs. 24.7±1.4 ng/mg protein; OVCA429/NICD3 cells, 26.8±2.9 vs. 25.1±1.4 ng/mg). ROS can contribute to the killing effect of MSeA and carboplatin, but are not required for the synergism in OVCA429/NICD3 cells. Because serum selenium and carboplatin concentrations have been reported to amount 15 and 105 µmol/L, respectively [46], [47], the doses of MSeA and carboplatin employed in this study are clinically relevant and pharmacologically achievable. Nonetheless, it is of future interest to confirm the synergism in other Notch3-activated cancer cells and in pre-clinical and clinical settings.

*HES1* and *HEY1* are classical NICD3 target genes. HES1 is a transcriptional repressor whose roles in the Notch signaling pathway have begun to be appreciated [30], [48]. NICD3 transactivates *HES1* expression by dissociating its co-repressors and allowing co-activators to bind. As a transcriptional repressor, HES1 subsequently can influence cell proliferation. Although HES1 up-regulation is known to promote carcinogenesis [49], there are numerous reports demonstrating down regulation of HES1 in association with prostate cancer progression [50] and in aggressive tumors [51]. Since MSeA induces the mRNA expression of *HES1* but not *HEY1*, MSeA may not directly act on NICD3. Furthermore, MSeA induces a greater *HES1* mRNA expression in OVCA429/pCEG than in OVCA429/NICD3 cells, suggesting that MSeA can stimulate the binding of co-activators independent of NICD3. It is also likely that Notch1 and Notch2 account for the significant HES1 induction in OVCA429/pCEG cells. The differential regulation of MSeA and carboplatin on the mRNA expression of the NICD3 target genes may at least partially explain the synergism in OVCA429/NICD3 cells. However, because treatment with the same doses of MSeA and carboplatin instead result in antagonism in OVCA429/pCEG cells, cautious consideration should be taken pertaining the cell-specific and dose-dependent nature of the synergism. This notion is consistent with the understanding that the range of effective selenium chemoprevention is narrow and cancer-specific [39]. Furthermore, MSeA treatment may promote the expression of some cancer-promoting selenoproteins in OVCA429/pCEG cells [52]. Future studies are needed to elucidate this MSeA-induced transcriptional regulation and to verify the role of HES1 and other Notch target genes in pre-clinical models and clinical samples of ovarian cancer.

Low percent S phase population, as displayed in OVCA429/NICD3 cells, is indicative of rapid DNA replication and poor response to chemotherapy [53], [54]. After the MSeA and carboplatin co-treatment, the percent S phase population drops in OVCA429/pCEG cells whereas the percent G2/M population declines in OVCA429/NICD3. The declines amount similarly to the increased sub G1 population, suggesting that the co-treatment may target S phase in OVCA429/pCEG cells and G2/M phase in OVCA429/NICD3 cells for apoptosis. It is possible that the replication stress and DNA breaks induced by carboplatin and MSeA sensitize OVCA429/pCEG cells in the S phase of the cell cycle, but homologous recombination is activated in OVCA429/NICD3 cells. As such, this would require a combinational treatment to induce mitotic stress and target G2/M phase OVCA429/NICD3 cells to death. Although a combination of gallate and sulforaphane sensitizes advanced stage ovarian cancer cells to cisplatin treatment through G2/M arrest [55], the MSeA and carboplatin co-treatment appears to target S phase OVCA429/pCEG cells and G2/M phase OVCA429/NICD3 cells.

Antioxidant therapy has been proposed to reduce the side effects in association with carboplatin treatment including ototoxicity, nephrotoxicity, and gastrointestinal toxicity [56]-[59]. However, the impact of oxidative or reductive stress on the treatment of ovarian cancer is not clear. Because NAC treatment desensitizes OVCA429 ovarian cancer cells to carboplatin and MSeA co-treatment independent of NICD3 expression, oxidative stress appears to be a general but not a specific requirement for effective suppression of ovarian cancer cell growth. Consistent with our observation, NAC has been shown to inhibit cisplatin-induced apoptosis in both lung and ovarian cancer cells [56].

It is intriguing that the MSeA and carboplatin co-treatment differentially activates ATM and DNA-PK_cs_ kinases, but inhibition of their kinase activities does not impact on the synergism of MSeA and carboplatin in killing the ovarian cancer cells. After the co-treatment, ROS appear to be required for the activation of ATM but contribute only partially to DNA-PK_cs_ kinase activation in OVCA429/pCEG cells. However, ATM kinase is not activated and DNA-PK_cs_ is activated independent of NAC in OVCA429/NICD3 cells. Although ATM is upstream of DNA-PK_cs_ in the response of MSeA-induced oxidative stress in non-cancerous cells [39], DNA-PK_cs_ activation by the co-treatment in OVCA429/NICD3 cells seems to be independent of ATM activation. Because DNA-PK_cs_ activation can sustain intracellular oxidative stress after MSeA treatment [39], the expression of NICD3 may predispose the cells to oxidative stress. On the other hand, although ATM kinase is activated by oxidative stress in HCT116, PC-3 and U2-OS cancer cells [12]-[14], it is striking that ATM kinase cannot be activated in OVCA429/NICD3 cells after the co-treatment. Since ovarian cancer stem cells express high levels of ATM [22], the role of ATM in carboplatin-resistant ovarian cancer awaits further verification.

Ovarian cancer is the fifth-leading cause of cancer death among women in the United States. Because most patients are diagnosed at advanced stage due to invalidated screening test and non-specific symptoms presented, they need a combination of debulking surgery and chemotherapy. However, patients typically have recurrent cancer following treatment and even develop chemoresistance. Thus, overcoming the resistance to chemotherapy is one of the promising strategies to treat ovarian cancer. In addition to the combinational effect of MSeA with paclitaxel, curcumin, or ABT-737 in the apoptotic death of prostate and breast cancer cells [60]-[62], our data provide direct support for a synthetic lethal interaction between MSeA and carboplatin in ovarian cancer cells expressing NICD3 and exhibiting chemoresistance. In conclusion, MSeA and carboplatin synthetic lethality is a promising prospect for late stage ovarian cancer therapy.

## Supporting Information

File S1 (PDF)
