# Supplementary material for: Methylseleninic Acid Sensitizes Notch3-Activated OVCA429 Ovarian Cancer Cells to Carboplatin
Source: PLoS One. 2014 Jul 10;9(7):e101664. doi: 10.1371/journal.pone.0101664 (PMC4092030; doi:10.1371/journal.pone.0101664)
Supplement: File S1 — (PDF) [file pone.0101664.s001.pdf]

Supplemental Table 1. Sequences of primers for the quantitative RT-PCR analyses (human)

Gene	Forward (5' to 3')	Reverse (5' to 3')
HEY1	TTTCGGCTCCTTCCACTTAC	CCTCCCTCATTCTACATCAGTTC
HES1	GTCAACACGACACCGGATAA	TTCAGCTGGCTCAGACTTTC
β-actin	GGACCTGACTGACTACCTCAT	CGTAGCACAGCTTCTCCTTAAT

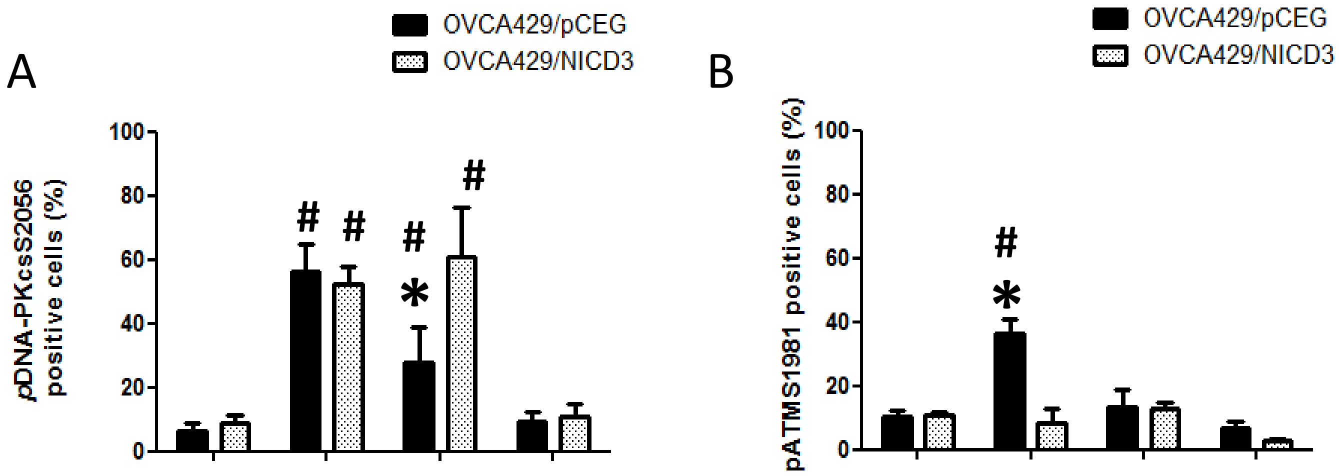

A

**MSeA + Carboplatin**	-	+	+	+
**NAC**	-	-	+	-
**NU 7026**	-	-	-	+

B

**MSeA + Carboplatin**	-	+	+	+
**NAC**	-	-	+	-
**KU 60019**	-	-	-	+

Figure S1. Immunofluorescent analyses of pDNA-PK$_{cs}$ S2056 (A) and pATM S1981 (B) expression in OVCA429/pCEG and OVCA429/NICD3 cells co-treated with MSeA (2 µmol/L**) and carboplatin (5** µmol/L**) for 24 h.** Some cells were co-treated with NU 7026 (10 µmol/L), KU 60019 (10 µmol/L), or NAC (10 mmol/L). Values are mean ± S.E.M. (n = 3). *, p < 0.05, compared to OVCA429/NICD3 cells. #, *p* < 0.05, compared to no treatment.

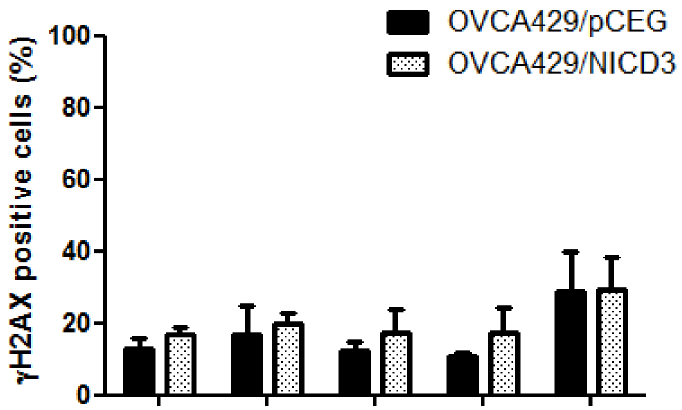

**MSeA + Carboplatin**	-	+	+	+	+
**NAC**	-	-	+	-	-
**NU 7026**	-	-	-	+	-
**KU 60019**	-	-	-	-	+

Figure S2. Immunofluorescent analyses of γH2AX expression in OVCA429/pCEG and OVCA429/NICD3 cells co-treated with MSeA (2 μmol/L**) and carboplatin (5** μmol/L**) for 24 h.** Some cells were co-treated with NU 7026 (10 μmol/L), KU 60019 (10 μmol/L), or NAC (10 mmol/L). Values are mean ± S.E.M. (n = 3).
